# Eye-Head Coordination in 31 Space Shuttle Astronauts during Visual Target Acquisition

**DOI:** 10.1038/s41598-017-14752-8

**Published:** 2017-10-27

**Authors:** Millard F. Reschke, Ognyan I. Kolev, Gilles Clément

**Affiliations:** 1 Neuroscience Laboratories, NASA Johnson Space Center, Houston, TX USA; 2 University Hospital of Neurology and Psychiatry, Sofia, Bulgaria; 3 Lyon Neuroscience Research Center, Bron, France; 4 KBRwyle, Houston, TX USA

## Abstract

Between 1989 and 1995, NASA evaluated how increases in flight duration of up to 17 days affected the health and performance of Space Shuttle astronauts. Thirty-one Space Shuttle pilots participating in 17 space missions were tested at 3 different times before flight and 3 different times after flight, starting within a few hours of return to Earth. The astronauts moved their head and eyes as quickly as possible from the central fixation point to a specified target located 20°, 30°, or 60° off center. Eye movements were measured with electro-oculography (EOG). Head movements were measured with a triaxial rate sensor system mounted on a headband. The mean time to visually acquire the targets immediately after landing was 7–10% (30–34 ms) slower than mean preflight values, but results returned to baseline after 48 hours. This increase in gaze latency was due to a decrease in velocity and amplitude of both the eye saccade and head movement toward the target. Results were similar after all space missions, regardless of length.

## Introduction

In 1989, the National Aeronautics and Space Administration (NASA) began a program to progressively lengthen Space Shuttle missions from 4 days to 17 days, and plans included missions of up to 28 days. Part of this program included a series of investigations to assess crew performance during and after the critical phases associated with Space Shuttle landings. Using data obtained during the first 24 Space Shuttle missions, investigators determined that the primary concerns were orthostatic intolerance and neurovestibular deficiencies^1^. Despite intensive simulator training, some Space Shuttle commanders and pilots were unable to land the Shuttle with the desired performance specifications after short (less than 8 days) missions^2^. Because the crew would continue to land the vehicle manually, keeping the autoland capability for an emergency backup only, landing proficiency was anticipated to degrade even more after extended Space Shuttle missions. However, no data was available to determine whether changes in landing proficiency might be related to the length of time spent in the microgravity environment^1^.

The objective of the present study was to assess how astronauts perform a visual task that was required to pilot and land the Space Shuttle. We measured the eye and head movements of pilots as they acquired specific visual targets in the horizontal plane before and after they participated in a Space Shuttle mission of 6 to 17 days. If pilots are unable to adapt their eye-head coordination to the changes in gravitational environment they will have difficulty acquiring information from instrumentation or will have delays capturing visual targets. The risk is greater in situations that require constant vigilance, timely responses, and accurate visual target identification and/or location, such as during a Space Shuttle landing. On final approach for landing, the Space Shuttle’s speed exceeded 300 knots, and it descended nearly 53 m in altitude and traveled more than 145 m downrange during the time required for the pilot to acquire a single target displaced 30° to 60° from straight ahead. Therefore, there was concern that a delay in visual target acquisition might compromise the safety of crews on short-term as well as extended-duration flights^3^.

Previous reports have documented modifications in eye-head coordination during visual target acquisition and decreases in ocular saccadic performance in astronauts after they return from short duration spaceflights^4–8^. However, for these previous studies, data was collected one or two days after landing and the studies involved a very small number of participants. We were fortunate to collect data just a few hours after landing (on R + 0) from 31 astronauts, most of whom were Space Shuttle pilots. This larger number of participants allowed us to assess whether a correlation existed between the astronauts’ performance and the duration of their spaceflight. The results of our study provide a complete picture of the difficulties astronauts face when attempting to acquire specific visual targets after returning from short-duration spaceflight.

## Methods

### Participants

A total of 31 healthy human subjects (29 male, 2 female) participated in the study; their mean age was 41.1 years (*SD* = 5.9), with ages ranging from 33 to 58 years. The subjects were Space Shuttle commanders, pilots, or mission specialists with professional piloting experience. The NASA institutional review board for human testing approved all test procedures. The methods were carried out in accordance with the relevant guidelines and regulations. Written informed consent was obtained from all participants before testing began.

### Study design

**Figure 1:**
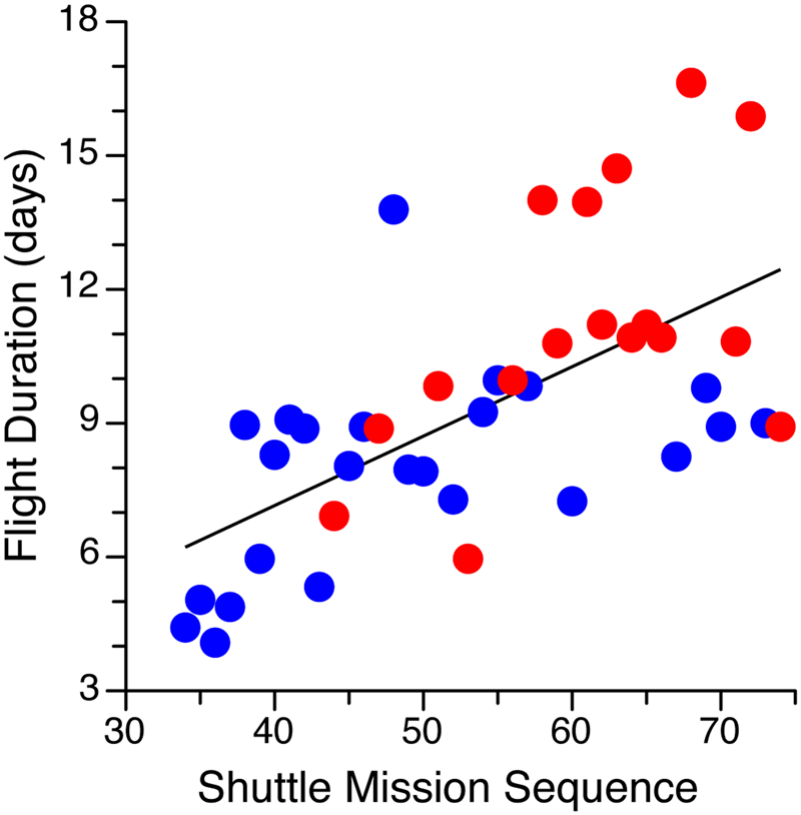
The NASA Extended Duration Orbiter Program included 41 Space Shuttle missions, beginning with the 33^rd^ Space Shuttle mission. Mission duration generally increased over time from 4 days to 17 days (blue and red symbols represent the individual missions). The results reported in the present paper were obtained from individuals who participated in 17 Space Shuttle missions lasting from 6 to 17 days (red symbols).

Subjects participated in one of 17 Space Shuttle missions lasting 6 days to 17 days. Eight subjects participated in one of 6 missions lasting from 6 to 9 days (mean 8.4); 14 subjects participated in one of 6 missions lasting from 10 to12 days (mean 11.0); and 9 subjects participated in one of 5 missions lasting from 14 to17 days (mean 15.0). As shown in Fig. 1, flight duration increased progressively during the Extended Duration Orbiter Program, starting with STS-36 (4.5 days) and culminating with STS-72 (17 days). The investigation presented here began about 18 months into the program and was conducted from November 1991 to January 1996.

Our visual target acquisition experiment was one of 4 protocols to investigate changes in visual-vestibular interactions as a function of exposure to spaceflight: visual target acquisition, gaze stabilization, pursuit tracking, and sinusoidal oscillations^3^. The crewmembers were tested at 3 different times before flight and 3 different times after the flight. Preflight testing took place at the NASA Johnson Space Center 120 days before launch, 30 days before launch, and 10 days before launch. Early postflight testing was performed on the landing site (either at NASA Kennedy Space Center or at NASA Dryden Space Research Center) within 2 hours of wheels stop (R + 0). Postflight testing was performed again at NASA Johnson Space Center 2 days after landing (R + 2) and 4 days after landing (R + 4) to track recovery to baseline.

### Equipment

Visual targets subtending 0.5° were fixed to a screen on a horizontal plane at 20°, 30°, and 60° angular distances to the right and the left of the subject’s straight-ahead view. The distance between the screen and the subjects was 75 cm. To easily differentiate between targets, each target had a different color according to the degree of angular offset from center. The subjects were instructed to use both their head and eyes to move their gaze as quickly and accurately as possible from the central fixation point to a specified target indicated by the operator. The order of the specified targets was randomly predefined. After each visual target acquisition the subjects returned their gaze to the center fixation position. During each test session, data was collected a minimum of 3 and a maximum of 5 times for each of the 6 target locations.

Horizontal eye movements were recorded with non-polarizing ECG electrodes positioned at the outer canthus of each eye, with a reference electrode located on the mastoid process behind the right ear. Head movements were recorded with a triaxial rate sensor system mounted on a headband that was attached firmly to the subject’s head. Eye movements were calibrated by having the subjects look at the same visual targets but without moving their head. Analog signals from the eye electrodes and rate sensors were amplified and digitized at a sampling rate of 500 Hz. To remove extraneous high-frequency noise, the measured wave forms were digitally filtered before being processed with a finite impulse response, low-pass Hamming window filter with a nominal cutoff frequency (−3 dB point) of 30 Hz. Data were passed through the filter twice—once forward in time and once backward in time to eliminate all phase shifts and double the stop-band attenuation.

### Data analysis

The eye position signal was differentiated to calculate eye velocity. The rate sensor signals were integrated to calculate head movement amplitude around the yaw, pitch, and roll axes. During visual target acquisition the head movement was almost exclusively around the yaw axis.

Responses for acquiring the target at each location were averaged for each subject. Additionally, all of the preflight measurements were averaged for each subject to provide a single preflight value for each target eccentricity against which the subsequent postflight data could be compared.

Shapiro-Wilk tests were used to analyze the normality of each variable. The peak velocity and peak amplitude of the primary eye saccade and head movement were analyzed separately using analysis of variance (ANOVA). Two-way repeated-measures ANOVAs were conducted to compare the effects of test sessions (Pre, R + 0, R + 2, R + 4) and target positions (20°, 30°, 60°). Post hoc analyses to compare the preflight session and the postflight sessions used Bonferroni correction to reduce Type I error.

### Data availability

All data generated or analyzed during this study are included in this published article.

## Results

### Eye-head coordination

**Figure 2:**
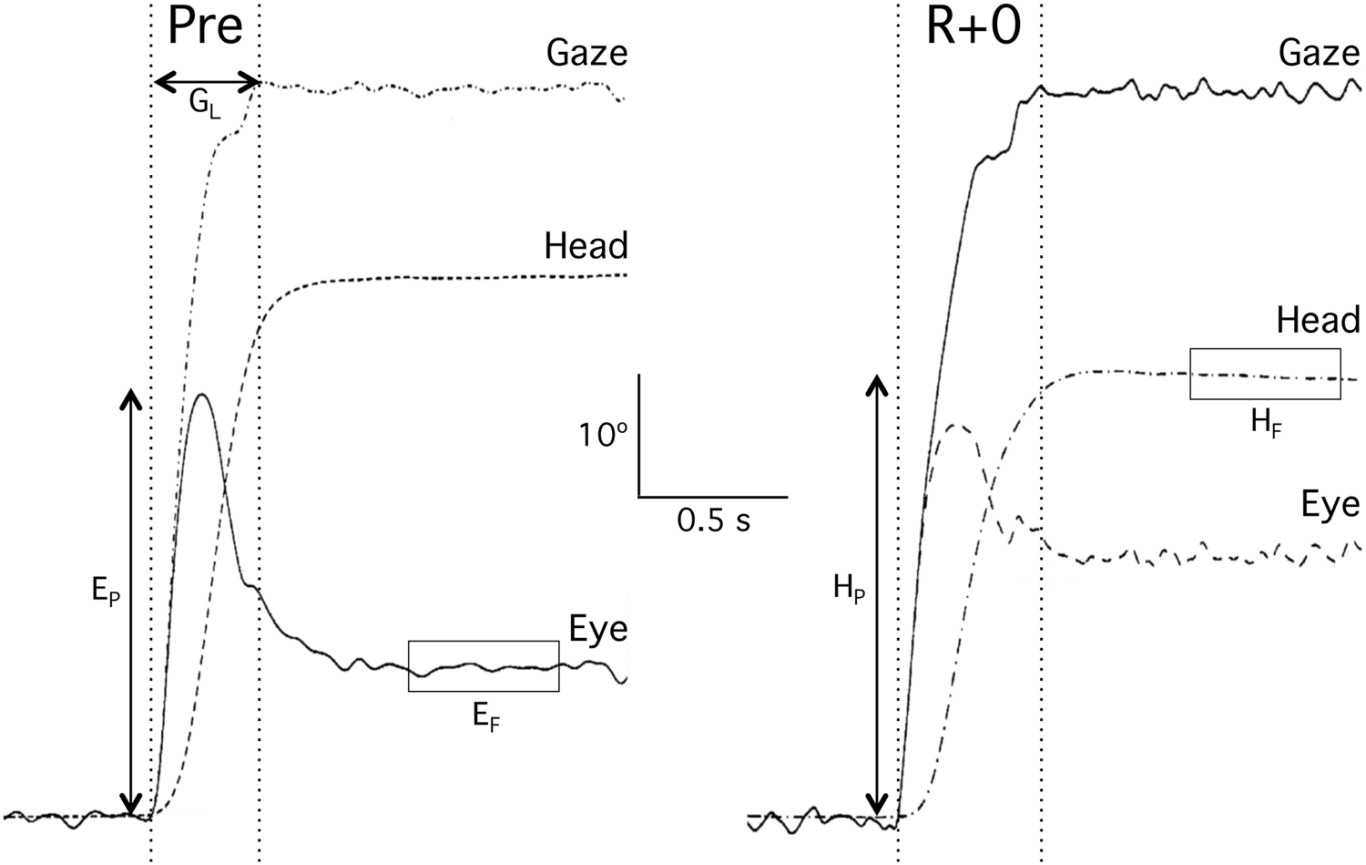
Recordings of eye, head, and gaze (sum of eye and head) positions during visual acquisition of a target 60° off center, obtained before (Pre) and immediately after (R + 0) the astronaut returned from a 16-day Space Shuttle mission. The dependent measures included the gaze latency (G_L_); the amplitude of the primary eye saccade (E_P_); the amplitude of head rotation (H_P_); and the final position of the eye (E_F_) and head (H_F_) averaged over a period of 0.5 s starting 0.5 s after the gaze was on target^9^.

While acquiring images 20° and 30° off center in the horizontal plane, the eyes and the head make one single movement to the target. The head, having greater inertia than the eye, typically moves after the eye has moved in the orbit. Gaze is the direction of the visual axis with respect to space, which is defined as the sum of eye position with respect to the head, and head position with respect to space. At the end of the eye saccade the gaze has already reached its final position, but the head continues to move. The head movement stimulates the horizontal semicircular canals and produces an eye movement through the vestibulo-ocular reflex that is opposite in direction and velocity to that of the head. When moving to a target that is offset 60°, the gaze often undershoots the target by 5–10° and a corrective eye saccade occurs to reposition the gaze onto the target. In general, the total gaze movement is greater than the total head movement, so that the final position of the eye is offset in the direction of head movement. Comparison of the recordings obtained before and immediately after flight indicated that the amplitudes of the initial eye saccade and head movement were smaller and the duration of gaze movement to the target was longer after flight than before flight (Fig. 2).

### Gaze latency

**Figure 3:**
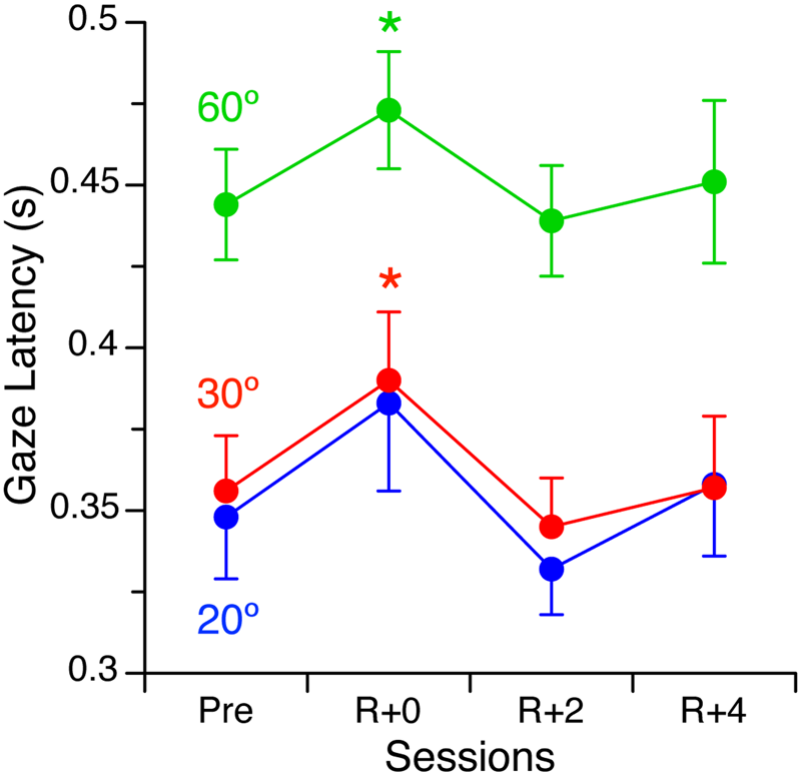
Gaze latency during visual acquisition of targets at 20°, 30°, and 60° off center before flight (Pre), immediately after landing (R + 0), and 2 days (R + 2) and 4 days (R + 4) later. Mean ± standard error of 31 subjects. *P* < *0.05 relative to Pre.

The delay between the start of the eye and head movements after flight (R + 0: 24.9 ± 32.4 ms) was not significantly different from the delay before flight (19.4 ± 25.6 ms), when the subjects took 350 to 440 ms to reach the target. This gaze latency increased as a function of the angular position of the target (Fig. 3). The effects of spaceflight on gaze latency were analyzed using a 3 targets (20°, 30°, 60°) × 4 sessions (Pre, R + 0, R + 2, R + 4) repeated-measures ANOVA, alpha = 0.05. There was a significant effect of target position [F (2,360) = 29.74, P < 0.001] and a significant effect of spaceflight [F (3,360) = 2.76, P = 0.04] on gaze latency, but no significant interaction between target position and spaceflight [F (6,360) = 0.05, P = 0.99]. An increase in gaze latency was observed in more than two-thirds of the subjects on R + 0. The largest increase relative to preflight latency was 172 ms. When averaged across all subjects, the gaze latency for reaching the 30° and 60° targets increased relative to preflight latency by 34 ms (P = 0.024) and 30 ms (P = 0.006), respectively. Responses had returned to baseline on R + 2.

### Eye and head velocity

**Figure 4:**
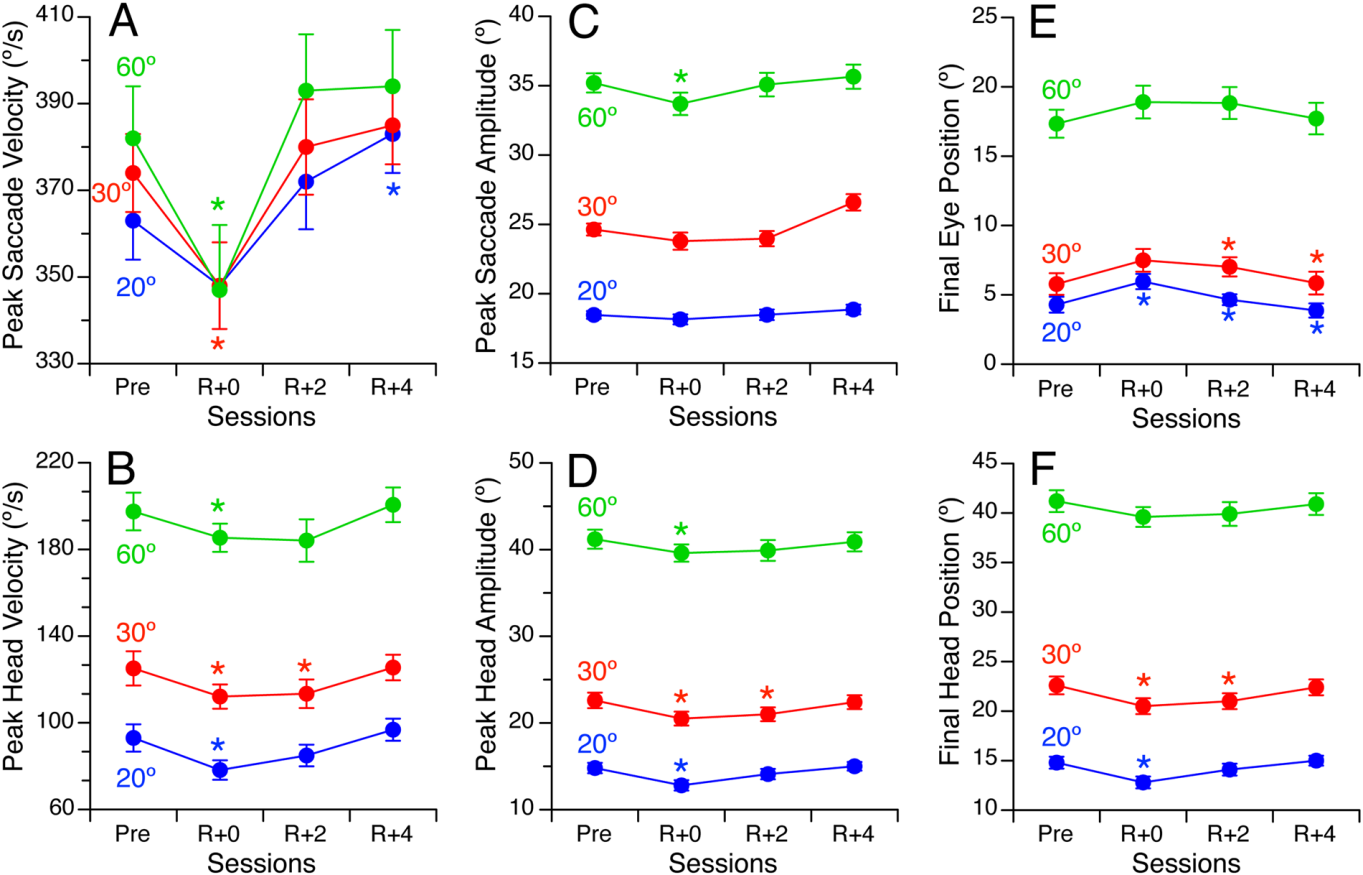
Measurements of eye and head movements during visual acquisition of targets at 20°, 30°, and 60° off center before flight (Pre), immediately after landing (R + 0), and 2 days (R + 2) and 4 days (R + 4) later. Mean ± standard error of 31 subjects. *P < 0.05 relative to Pre. (**A,B**) Peak velocity of the eye primary saccade and head movement. (**C,D**) Amplitude of the eye primary saccade and head turn. (**E,F**) Final position of the eye in the orbit and the head in space.

The time to bring gaze on target is determined by the maximal eye and head velocities^9^. Before the flight the mean peak velocities of the initial eye saccade were 363.4°/s (SD = 54.7), 374.6°/s (SD = 60.7), and 382.3°/s (SD = 68.1) for the targets at 20°, 30°, and 60° respectively (Fig. 4A). A two-way repeated-measure ANOVA indicated a significant effect of spaceflight [F (3,360) = 6.91, P < 0.001] on peak eye velocity, and no difference between the targets [F (2,360) = 1.02, P = 0.36]. On R + 0, the peak eye velocity when acquiring for the 30° and 60° targets decreased relative to preflight peak velocity by 26.0°/s (P < 0.01) and 36.4°/s (P < 0.01), respectively. Responses had returned to baseline on R + 2.

The peak head velocity was related to position of the target; the more offset the target, the faster the head movement (Fig. 4B). A two-way repeated-measure ANOVA indicated a significant difference in peak head velocity between the targets [F (2,360) = 256.3, P < 0.001] and a significant effect of spaceflight [F (3,360) = 4.50, P < 0.01]. On R + 0, the peak head velocity when acquiring the targets at 20°, 30°, and 60° decreased relative to preflight peak velocity by 10.0°/s (P < 0.01), 6.9°/s (P < 0.02), and 6.7°/s (P < 0.05), respectively. On R + 2, the peak head velocity was still significantly slower than preflight peak velocity (P < 0.02) when acquiring the 30° targets, whereas the responses for acquiring the other targets had returned to baseline.

### Eye and head amplitude

Repeated-measures ANOVA indicated a significant effect of target position on the peak amplitudes of the eye saccade [F (2,360) = 864.8, P < 0.001] and head movement [F (2,360) = 1067, P < 0.001]). Spaceflight affected the peak amplitude of the head movement [F (3,360) = 4.38, P < 0.01], but not the peak amplitude of the eye saccade (Fig. 4C). Relative to preflight values, the head amplitude decreased on R + 0 by 1.5°–2.2° (P < 0.05) depending on the target offset (Fig. 4D).

During both preflight and postflight tests, the gaze displacement remained essentially constant after the initial eye saccade ended (Fig. 2), indicating that the slow-phase eye compensation was very precise. During this phase of the response, the vestibulo-ocular reflex gain (ratio of eye velocity and head velocity) was near unity across all targets and test sessions. In addition, the number and amplitude of the corrective saccades at the end of the head movement toward the 60° targets were not significantly affected by spaceflight.

Repeated-measures ANOVA indicated an effect of spaceflight on the final position of the eye in the orbit [F (3,360) = 3.16, P < 0.03] (Fig. 4E) and on the final position of the head in space [F (3,360) = 3.51, P < 0.02] (Fig. 4F). Post-hoc tests revealed that the final eye position during acquisition of the 20° target was significantly larger on R + 0 (P < 0.01), R + 2 (P < 0.001), and R + 4 (P < 0.001) compared to preflight. This effect was also observed during acquisition of the 30° target on R + 2 (P < 0.01) and R + 4 (P < 0.01). The final head position during acquisition of the 20° target was significantly smaller than preflight on R + 0 (P < 0.001). This effect was also observed during acquisition of the 30° target on R + 0 (P < 0.001) and R + 2 (P < 0.001).

### Effect of flight duration

**Figure 5:**
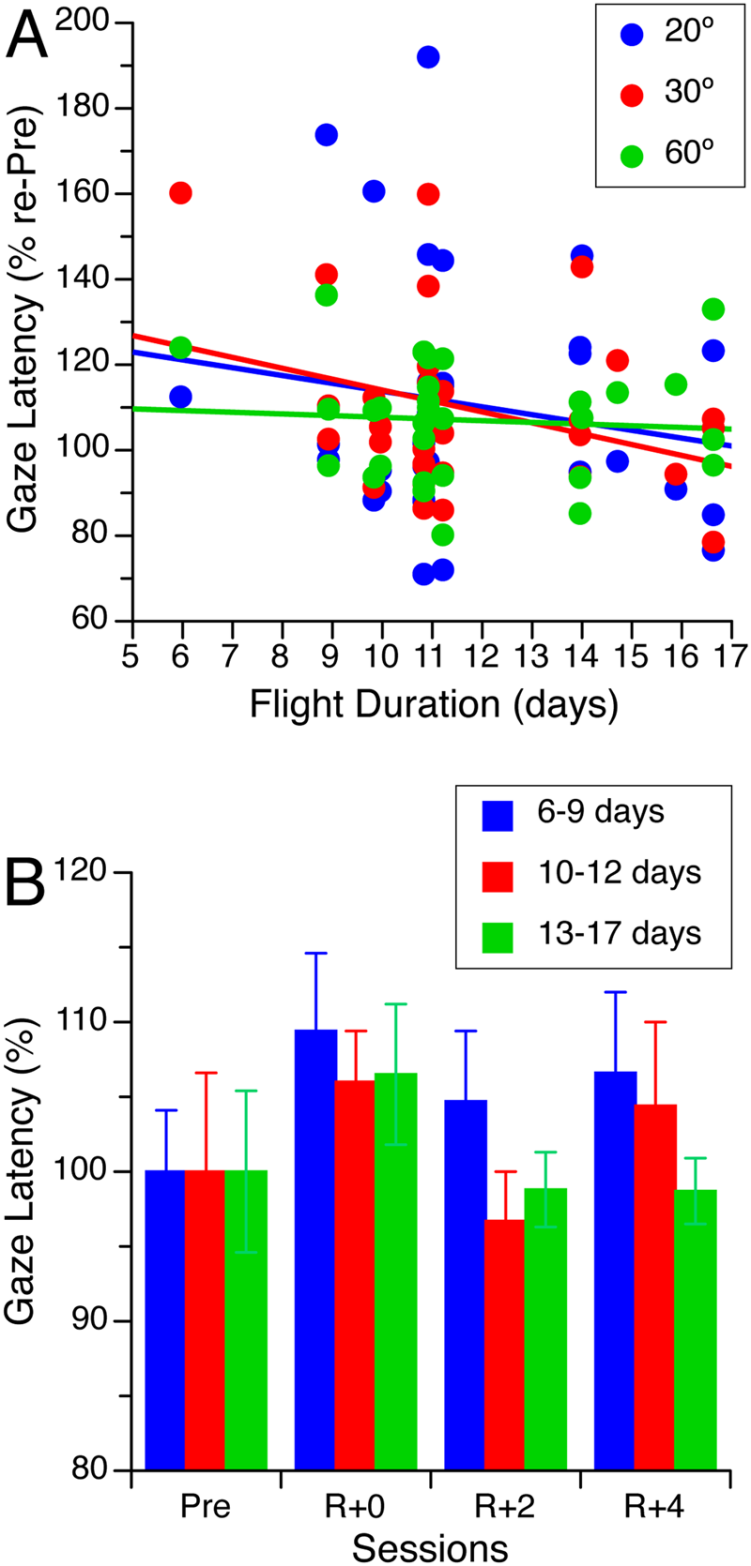
(**A**) Changes in gaze latency on R + 0 relative to preflight (% re-Pre) for each visual target and each of the 31 crewmembers. (**B**) Gaze latency during visual acquisition of the target at 60° off center after space missions of short (6–9 days, N = 8), medium (10–12 days, N = 14), and long (13–17 days, N = 9) durations. This comparison of gaze latencies across flight duration focuses on the 60° target since the overall increase in gaze latency after flight was more robust for this target (see Fig. 3). Responses are expressed in percentages of the baseline (Pre) measurements.

Figure 5A indicates that the increase in gaze latency immediately after landing (see Fig. 3) was not correlated with the duration of the flight. Each subject’s responses during visual target acquisition after spaceflight were normalized for each visual target according to their mean gaze latency before flight. Individual responses were then grouped by flight duration into 3 categories (short: 6–9 days; medium: 10–12 days; long: 13–17 days), and the group mean was calculated for each test session. The effects of spaceflight duration on the gaze latency were analyzed using repeated-measures ANOVA with 3 targets (20°, 30°, 60°) × 3 durations (short, medium, long). There was no significant effect of target [F (2,360) = 0.23, P = 0.79] or spaceflight duration [F (2,360) = 1.41, P = 0.26] on gaze latency. Figure 5B illustrates mean gaze latencies for acquiring the 60° visual target (group average) after short-, medium-, and long-duration space missions.

## Discussion

The results of this study indicate that the time to acquire a visual target located 30° and 60° off center in the horizontal plane increased by 7–10% (30–34 ms) during the first few hours after return form space. This could potentially result in piloting errors because the astronauts will experience delays capturing operationally relevant targets during the critical period when they are returning to Earth. Time delays to acquire visual targets were consistent after short- (6–9 days), medium- (10–13 days), and long- (14–17 days) duration Space Shuttle missions. Responses returned to normal 48 hours after landing. The increase in gaze latency was attributed to a decrease in velocity and amplitude of both the initial eye saccade and head movement toward the target.

Data on acquiring horizontal visual targets during spaceflight are rare and results are conflicting. Some researchers have reported increased latencies of eye saccade and decreased peak velocities during flight^8^, whereas others have reported opposite effects^6^. The studies that showed diminished performance were conducted early in flight and often involved motion-sick subjects, whereas the study by Uri *et al*.^6^, reporting enhanced performance, was conducted on the seventh day in space. Horizontal-pursuit eye movements and the horizontal vestibulo-ocular reflex gain during active or passive head oscillations in yaw were unchanged during spaceflight^10–16^. By contrast, spaceflight did affect vertical-pursuit eye movements and the vertical vestibulo-ocular reflex gain^8,14,15^. Upward pursuit was accomplished primarily by saccades and downward pursuit by a combination of saccades and smooth eye movements^17^. Gaze behavior quickly readapts after these flights, often resolving within one day of return to Earth.

Longer spaceflights on board Mir and the International Space Station have allowed time for substantial adaptation to the 0 *g* environment, and astronauts who have participated in these missions have had decreases in performance that persisted for several days after landing^5,7,18^. Changes relative to preflight measurements were observed in vertical smooth pursuit and gaze stabilization during head and eye movements in the vertical plane^4,19–21^. Responses in the horizontal plane were not affected. However, in these studies the earliest postflight measurements took place one or two days after the landing of the Soyuz capsule in Kazakhstan, so the responses in the horizontal plane could have already readapted. The present study is the first report of changes in acquiring horizontal visual targets with both eye and head movements in astronauts within a few hours of return from space.

Our subjects’ eye and head movements before spaceflight were comparable to eye movements that were measured in ground-based subjects using EOG and eye coil techniques^9,22,23^. Current models of eye-head coordination postulate that a vestibular signal serves as an integral component of saccadic spatial programming during head-free gaze shifts^22–26^. In these models, the desired gaze position is compared to an internal representation of the actual gaze position. This internal representation of gaze position is the sum of the perceived eye position in the head (derived from the efferent copy) and the perceived head position in space (derived from vestibular inputs). The difference between desired and actual gaze position produces a gaze position error signal that drives saccadic motor output until the error signal is cancelled (using visual feedback) and eye movement stops. The gaze movement is shared between the head and eyes, clearly a functionally advantageous manoeuver since the head and eyes are individually limited to lateral angular excursions of approximately ±75° and ±45° respectively^9^.

A number of investigators have assessed the role of vestibular-based subsystems during and immediately after spaceflight (see review in^27^). Several strategies persist as long as astronauts are in microgravity, including (1) reduced use of head movements, (2) reliance on an internal (intrinsic) coordinate system for spatial orientation, and (3) compensation for the changing role of proprioceptive information. Strategies that developed during spaceflight are still used immediately after return from spaceflight. If the newly acquired behavior is not appropriate, responses result in performance decrements, particularly in off-nominal situations^3^.

Because head movements provoke motion sickness during reentry, it is possible that the subjects reduced their head movements immediately after spaceflight to avoid discomfort. Also, the neck muscles could be weakened due to unloading of the head in microgravity. These factors could explain the increased latency for head movements and by association eye movements because the two systems are connected. However, pursuit eye movements that occur without head movements are also altered after spaceflight. These alterations have been attributed to changes in the tonic levels of otolith activity in the vestibulo-cerebellum^8^.

On Earth, the otoliths help the brain interpret the position of the head in space by detecting head tilt relative to gravity. In the absence of a gravitational reference during spaceflight, the static otolith signals are no longer effective, and visual and proprioceptive cues are primarily used to interpret the position of the head. During return to a 1*g* environment, otolith inputs are restored and proprioceptive information changes. Recent clinical studies suggest that altered vestibular and somatosensory inputs may lead to changes in an individual’s mental representation of space^28^. A misinterpretation of the head’s position in space could result in a misperception of gaze position, which would slow head and eye movement while acquiring visual targets. According to this hypothesis, the perceived direction of gaze is altered in astronauts immediately after spaceflight. To verify this hypothesis, we are currently conducting an investigation to determine if astronauts perceive straight-ahead direction differently after they return from spaceflight.

As indicated above, few studies have demonstrated a direct effect of spaceflight on saccade gain; however, ground-based studies have shown that spatial targeting of saccades may depend on the g vector. For example, on Earth eye saccades systematically tilt as a function of head tilt^29^, and directional errors of saccades to recollected targets increase during spaceflight^30^. Altered visually-guided saccades, vestibulo-ocular reflex, and eye-hand coordination strategies have also been observed during exposure to very brief periods (20 s) of microgravity and hypergravity during parabolic flight^31–33^. An important question is whether changes in eye-head coordination will also occur during short periods of reduced and enhanced gravity such as during sub-orbital flights^34^. Some commercial companies plan to fly space planes that will provide reduced gravity for 3–5 minutes and then land conventionally on a runway. The increase in gaze latency observed in the present study could therefore be a potential issue for the pilots of these sub-orbital flight vehicles during landing.
